# Albumin-globulin ratio and mortality in patients on peritoneal dialysis: a retrospective study

**DOI:** 10.1186/s12882-020-1707-1

**Published:** 2020-02-14

**Authors:** Fenfen Peng, Lingzhi Sun, Ting Chen, Yan Zhu, Weidong Zhou, Peilin Li, Yihua Chen, Yiyi Zhuang, Qianyin Huang, Haibo Long

**Affiliations:** Department of Nephrology, Zhujiang Hospital of Southern Medical University, Guangzhou, 510280 China

**Keywords:** Albumin-globulin ratio, Peritoneal dialysis, Mortality

## Abstract

**Background:**

Albumin-globulin ratio (AGR), a variable based on serum albumin and non-albumin proteins, has been demonstrated as a predictor of mortality in patients with malignant neoplasm. The aim of this study was to evaluate the prognostic value of AGR on peritoneal dialysis (PD) patients.

**Methods:**

We retrospectively analyzed 602 incident PD patients from January 1st, 2008, to December 31st, 2017, at our center and followed them until December 31st, 2018. Kaplan-Meier curves and multivariate Cox regression models were applied to analyze the association between AGR and all-cause of mortality and cardiovascular mortality.

**Results:**

The median follow-up time was 32.17 (interquartile range = 32.80) months. During follow-up, 131 (21.8%) patients died, including 57 patients (43.5%) who died due to cardiovascular diseases. Kaplan-Meier curves showed that patients with AGR > 1.26 had better rates of survival than those with AGR ≤ 1.25 (*p* < 0.001). After adjusting for potential confounders, the lower AGR level was significantly associated with an increased all-cause and cardiovascular mortality [hazard ratio (HR): 1.57, 95% confidence interval (CI): 1.07–2.32, *p* = 0.022 and HR: 2.01, 95% CI: 1.10–3.69, *p* = 0.023 respectively].

**Conclusions:**

Patients with a low AGR level had an increased all-cause and cardiovascular mortality. AGR may be a useful index in identifying patients on PD at risk for CVD and all-cause of mortality.

## Background

There is an increasing number of end-stage renal disease (ESRD) patients around the world. It was reported that the population of ESRD patients was more than 762,331 in 2016 according to the US Renal Data System 2018 Annual Data Report [1]. Peritoneal dialysis (PD) is an effective therapy for ESRD. Although there are some advantages of PD, the survival rate of PD patients remains inadequate. Hong Kong, which has the largest proportion of PD patients in the world, reported that the overall 5-years cumulative survival for PD patients was 50.7% in 2012 [2]; therefore, the risk factors associated with the mortality of PD patients are discommode.

The measured serum total protein includes albumin, globulin and prothrombotic proteins, prothrombin, fibrinogen, and other inflammatory proteins (e.g, C-reactive protein [CRP], interleukins, leukotrienes, and others) [3]. The albumin-globulin ratio (AGR), frequently reported in the comprehensive blood panel, calculated with the following equation: AGR = Albumin / [Total Protein-Albumin], is actually a reflection of all non-albumin proteins, which includes globulin and other prothrombotic proteins and inflammatory proteins [3].

Albumin functions include maintaining plasma colloid osmotic pressure, transporting substances, and acting as an antioxidant [4]. Serum albumin can reflect both inflammatory and malnourished status [5]. A low serum albumin level is a key characteristic of protein-energy wasting (PEW), which is common and an important risk factor for morbidity and mortality in patients on dialysis [6, 7]. Immunoglobulins, represent a major portion of the globulin involved in immunologic and inflammatory processes [8]. Inflammation was related to the prognosis of ESRD patients, that inflammatory cytokines and CRP were reported as strong predictors of a poor outcome in dialysis patients [9, 10]. Recent studies suggested that AGR was associated with adverse outcomes in patients with different types of cancer [11, 12] and some other diseases such as polyangiitis and heart failure [13, 14]. However, there was a lack of direct clinical evidence for the association of AGR and outcomes of PD patients. In this retrospective observational cohort study, we tested the hypothesis that AGR is a predictor of mortality in PD patients.

## Methods

### Study population

**Fig. 1:**
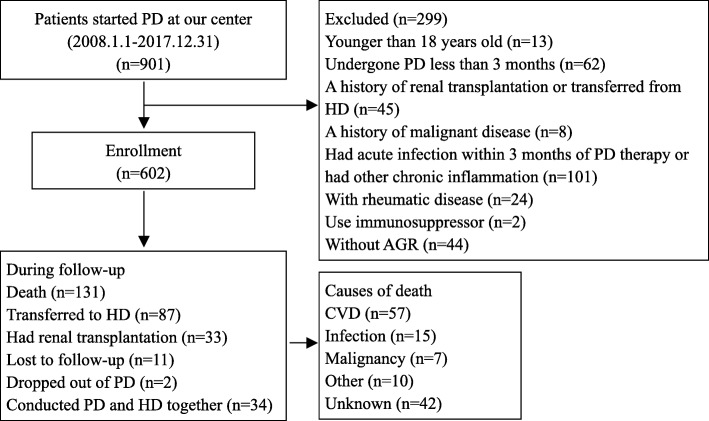
The flow chart of the study, including how patients selected and their outcomes. *PD* = Peritoneal dialysis, *HD* = Hemodialysis, *AGR* = Albumin-globulin ratio, *CVD* = Cardiovascular disease

This study employed 602 PD patients from January 1st, 2008 to December 31st, 2017 at the Zhujiang Hospital of Southern Medical University. The inclusion criteria were patients aged over 18 years old and under PD treatment for at least 90 days. Those who had any of the following characteristics were excluded: 1) patients transferred from hemodialysis (HD), renal transplantation or other centers; 2) patients with a malignant tumor before PD; 3) patients with liver cirrhosis, rheumatic disease, or consuming immunosuppressor medicine; 4) patients with acute infection within 3 months of PD therapy or had other chronic inflammation; 5) patients with insufficient data of AGR (Fig. 1).

### Clinical and laboratory data collection

The baseline data were collected during the first 1–3 months of PD. The total protein was measured through the biuret method and albumin was measured through the bromocresol green method in our hospital (Roche P800 and Mindray BS2000). The AGR was calculated by the following formula: AGR = Serum albumin / [Total serum protein-serum albumin] [11]. The definition of a diabetes patient was a patient who meets the diagnostic criteria of the American Diabetes Association [15]. A diagnosis of hypertension could be made if a patient was taking a hypotensor or their blood pressure ≥ 140/90 mmHg. The comorbidity score was assessed by the Charlson Comorbidity Index (CCI), and for each decade > 40 years of age, a score of 1 is added to the CCI score [16]. Total Kt/V was calculated using PD Adequest software 2.0 (Baxter Healthcare Ltd.). Average peritoneal dialysate glucose concentration (PDGC) was calculated as the total weight of glucose in the PD solution divided by the total solution volume, as previously reported [17].

The end point of the study was cessation of PD, death, or on December 31st, 2018, whichever came first. The primary outcome was all-cause mortality. The second outcome was cardiovascular mortality**,** which was defined as death caused by heart failure, cardiac arrhythmia, coronary arteriosclerotic heart disease, cardiomyopathy, congenital cardiovascular diseases, valvular heart disease, cardiac arrest, stroke, and peripheral vascular disease [18]. If a patient died in hospital, the death certificate was referred to for the exact cause of death. For those who died outside of the hospital, two clinical doctors made a decision about the cause of death, after a comprehensive review of the patient’s medical records and descriptions provided by the patient’s family members.

### Statistical analysis

IBM SPSS Statistics 20.0 software (SPSS Inc., Chicago, IL, USA) and R were applied to complete the statistical analysis. Data are described as number (percentage) for categorical variables, mean ± standard deviation for normally distributed variables, and median (inter-quartile range) for skewed distributed variables. Patients were divided into two groups according to the median of AGR. The characteristic differences between the baseline data of the two groups were compared using the T-test, Chi-square test, or Mann-Whitney U test, as appropriate. The association between AGR and clinical characteristics and laboratory data was analyzed using Spearman’s rank correlation analysis. Kaplan-Meier curves were performed to present the survival time of the two groups, and the differences were assessed by log-rank test. While taking the competitive risks of drop out due to causes other than death into consideration, Gray’s test was used to compare the difference of cumulative mortality incidence between the two groups using R. Covariates with *p* < 0.1 in the univariate Cox analyses or thought to be related to AGR levels were chosen for multivariate Cox regression model. *P* < 0.05 was considered to be statically significant. Power calculations were performed using PASS, version 11.0 for Windows.

## Results

### Baseline characteristics of patients

Six hundred-two PD patients met the inclusion criterion and were incorporated into this study, among these patients, 37 patients were on automated PD. Conventional PD solutions (Dianeal 1.5, 2.5 or 4.25% dextrose, Baxter Healthcare, Guangzhou, China) Y-set twin-bag systems were utilized in continuous ambulatory PD (CAPD) patients. The median of AGR was 1.25. Patients whose AGR ≤ 1.25 were entered into the low AGR group, and all others were included in the high AGR group. Baseline characteristics of the two groups are summarized in Table 1. The patients in the low AGR group were older, had a larger number of females and diabetes, as well as higher levels of CCI, leukocytes, serum globulin, platelet counts, red cell distribution width (RDW), and albumin-corrected calcium, but had a lower levels of serum albumin, phosphorus, creatinine, and urea nitrogen. The analysis of Spearman’s rank correlation showed that AGR level negatively correlated with age, CCI, total protein, leukocytes, platelet counts, RDW, and albumin-corrected calcium. In addition, AGR positively correlated with serum albumin, phosphorus, creatinine, and urea nitrogen. Serum albumin positively correlated with total protein, hemoglobin, serum creatinine, and urea nitrogen, and negatively correlated with age, CCI, and RDW. In addition, globulin positively correlated with total protein, age, CCI, leukocytes, platelet counts, RDW, and glucose, and also negatively correlated with serum creatinine and urea nitrogen (Table 2).

**Table 1:** The clinical characteristics of patients on peritoneal dialysis grouped by albumin-globulin ratio

Variable	Total	Lower AGR	Higher AGR	*p*
Demographics				
Gender (male, n, %)	356 (59.1%)	171 (54.8%)	185 (63.8%)	0.025
Age (y)	50.33 ± 14.63	53.88 ± 13.14	46.52 ± 15.20	< 0.001
Body mass index (kg/m^2^)	22.97 ± 3.32	22.99 ± 3.28	22.96 ± 3.38	0.915
Primary disease				
primary glomerular diseases(N,%)	349 (58.0%)	168 (53.8%)	181 (62.4%)	
Diabetic nephropathy(N,%)	91 (15.1%)	64 (20.5)	27 (9.3%)	
hypertensive nephropathy(N,%)	57 (9.5%)	28 (9.0%)	29 (10.0%)	
Obstructive nephropathy(N,%)	70 (11.6%)	37 (11.9%)	33 (11.4%)	
Other(N,%)	35 (5.8%)	15 (4.8%)	20 (5.9%)	
Comorbidities				
CVD (N,%)	153 (25.4%)	85 (27.2%)	68 (23.4%)	0.285
Diabetes (n,%)	141 (23.4%)	91 (29.2%)	50 (17.2%)	0.001
CCI Score	3.00 (3.00)	4.00 (2.00)	3.00 (2.00)	< 0.001
Hypertension(N,%)	556 (92.4%)	289 (92.6%)	267 (92.1%)	0.796
Laboratory variables				
Leukocytes(10^9^/L)	6.85 (2.34)	7.07 (2.71)	6.72 (2.21)	0.009
Hemoglobin (g/L)	97.13 ± 13.71	96.82 ± 13.52	97.47 ± 13.93	0.559
Platelet(10^9^/L)	236.20 ± 77.69	250.40 ± 82.03	220.87 ± 69.65	< 0.001
RDW(%)	14.95 (2.21)	15.25 (2.39)	14.73 (2.15)	< 0.001
Blood glucose (mmol/L)	4.50 (1.03)	4.52 (1.24)	4.49 (0.89)	0.154
Albumin-corrected calcium (mmol/L)	2.24 (0.21)	2.26 (0.20)	2.23 (0.20)	0.036
Phosphorus (mmol/L)	1.66 (0.62)	1.62 (0.53)	1.72 (0.67)	0.018
Serum Globulin (g/L)	26.47 ± 4.67	29.30 ± 4.07	23.42 ± 3.08	NA
Serm Albumin (g/L)	32.58 ± 4.22	30.89 ± 3.91	34.41 ± 3.77	NA
Cholesterol (mmol/L)	4.37 (1.01)	4.37 (1.14)	4.35 (0.92)	0.515
Triglycerides (mmol/L)	1.43 (0.96)	1.43 (0.97)	1.43 (0.91)	0.470
Serum uric acid (μmol/L)	432.12 ± 89.07	430.00 ± 88.57	434.41 ± 89.70	0.544
Serum creatinine (μmol/L)	792.84 (332.21)	758.25 (307.50)	832.77 (351.44)	< 0.001
Urea nitrogen (mmol/L)	19.50 (7.36)	18.91 (6.90)	20.14 (7.85)	0.002
24 h urine (ml)	846.31 (600.00)	846.31 (642.50)	846.31 (562.50)	0.562
Peritoneal dialysate volume(L/day)	7.97 ± 0.83	7.99 ± 0.87	7.95 ± 0.78	0.484
Total KT/V	2.15 (0.85)	2.20 (0.73)	2.06 (0.89)	0.058
PDGC(%)	1.50 (0.25)	1.50 (0.25)	1.50 (0.00)	0.002

Abbreviations: *AGR* Albumin-globulin ratio*, CVD* Cardiovascular disease, *CCI* Charlson comorbidity index, *RDW* Red cell distribution width, *PDGC* Peritoneal dialysate glucose concentration, *NA* Not applicable

**Table 2:** Correlation between baseline AGR and clinical, laboratory parameters

	AGR	Albumin	Globulin	Total protein	Age	CCI	Leukocytes	Hemoglobin	Platelet	RDW	Glucose	Phosphorus	Albumin-corrected calcium	Urea nitrogen
Albumin	0.518^b^													
Globulin	−0.759^b^	0.012												
Total protein	−0.149^b^	0.583^b^	0.722^b^											
Age	−0.319^b^	−0.305^b^	0.146^b^	−0.114^b^										
CCI	−0.299^b^	−0.331^b^	0.114^b^	−0.143^b^	0.804^b^									
Leukocytes	−0.133^b^	−0.054	0.150^b^	0.091^a^	0.073	0.081^a^								
Hemoglobin	0.077	0.189^b^	0.012	0.113^b^	0.009	0.028	0.071							
Platelet	−0.212^b^	0.016	0.257^b^	0.186^b^	0.112^b^	0.088^a^	0.421^b^	−0.029						
RDW	−0.186^b^	− 0.172^b^	0.092^a^	− 0.047	0.092^a^	0.044	0.076	−0.246^b^	0.022					
Glucose	−0.091^a^	− 0.04	0.089^a^	0.044	0.289^b^	0.351^b^	0.065	−0.093^a^	0.125^b^	0.011				
Phosphorus	0.144^b^	0.186^b^	−0.024	0.131^b^	−0.407^b^	−0.377^b^	− 0.011	−0.092^a^	− 0.026	0.042	− 0.189^b^			
Albumin-corrected calcium	−0.109^b^	− 0.045	0.073	0.012	0.076	0.07	−0.018	0.184^b^	0.132^b^	−0.097^a^	0.103^a^	−0.235^b^		
Urea nitrogen	0.174^b^	0.116^b^	−0.125^b^	0.001	−0.285^b^	−0.308^b^	− 0.090^a^	−0.179^b^	−0.145^b^	0.038	− 0.121^b^	0.609^b^	−0.139^b^	
Serum creatinine	0.210^b^	0.111^b^	−0.140^b^	0.011	−0.463^b^	−0.428^b^	− 0.011	−0.176^b^	−0.093^a^	0.059	− 0.214^b^	0.645^b^	−0.223^b^	0.574^b^

^a^ Correlation is significant at the 0.05 level (2-tailed)

^b^ Correlation is significant at the 0.01 level (2-tailed)

Abbreviations: *AGR* Albumin-globulin ratio, *CCI* Charlson comorbidity index, *RDW* = Red cell distribution width

### AGR and mortality

**Fig. 2:**
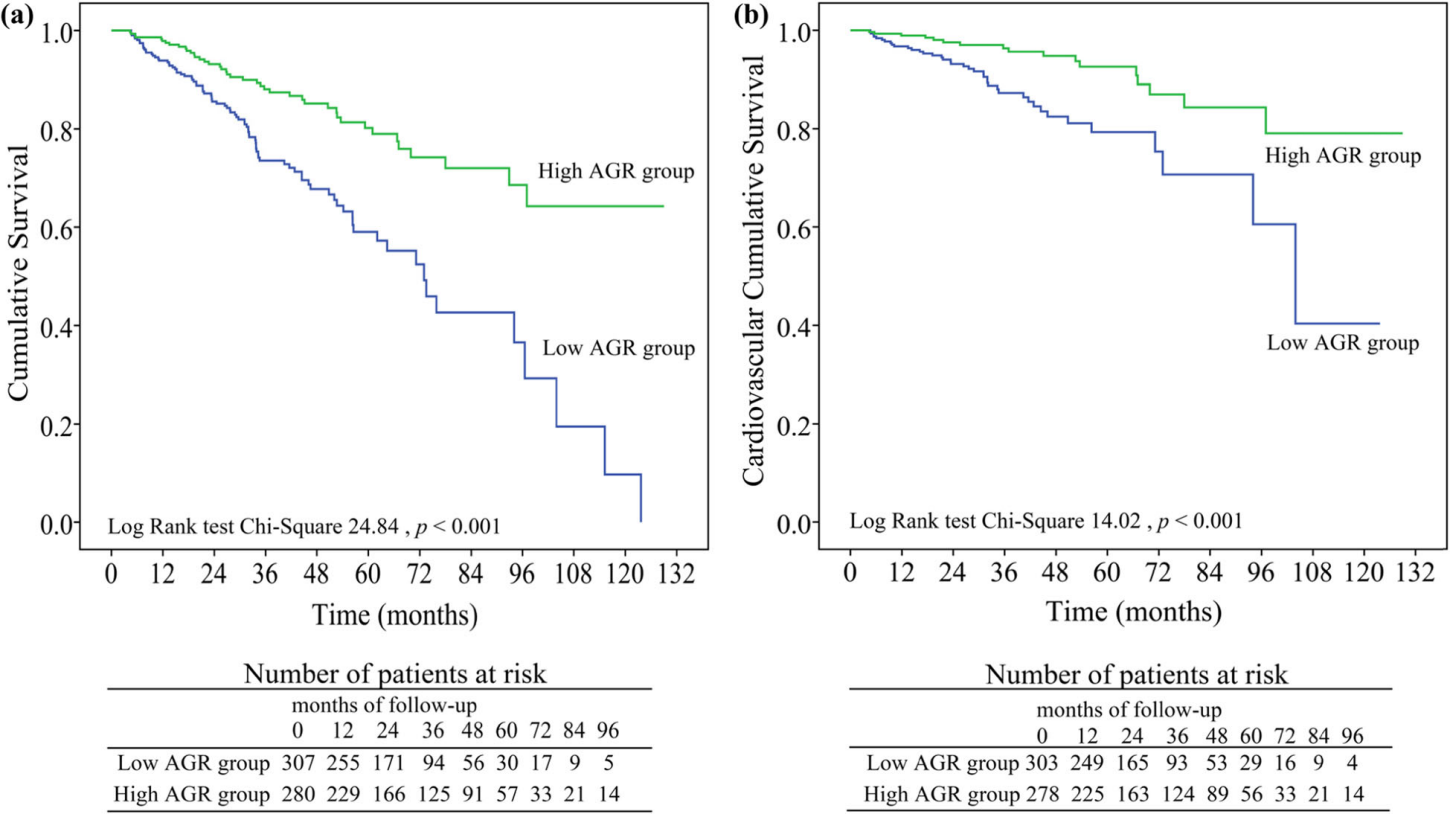
Crude analyses of all-cause and cardiovascular mortality between AGR groups. Cumulative mortality curves for (**a**) all-cause mortality, and (**b**) cardiovascular mortality

**Fig. 3:**
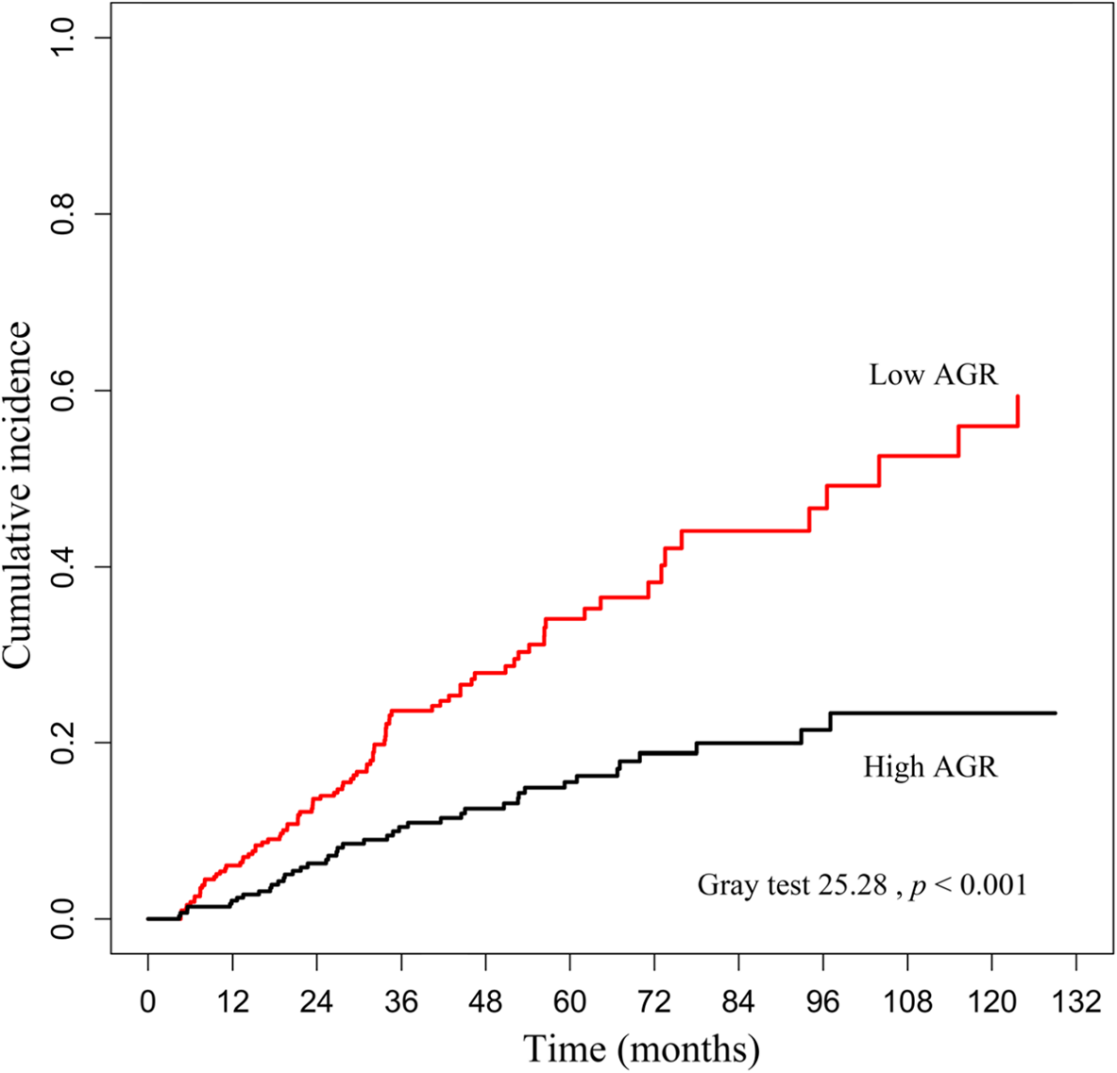
The cumulative incidence of mortality between AGR groups

For the entire cohort, the median follow-up time was 32.17 (interquartile range = 32.80) months. By the deadline of follow-up, 87 (14.4%) participants converted to hemodialysis; 33 (5.5%) patients had renal transplantation; and 11(1.8%) were lost to follow up. A total of 131 (21.8%) deaths occurred, 57 (43.5%) of which were caused by CVD (Fig. 1). At the end of 1, 3, 5, 7 and 10 years, the cumulative survival rate was 94, 74, 60, 44, and 11%, respectively, in the low AGR group and 98, 88, 81, 73, and 64%, respectively, in the high AGR group; the cardiovascular cumulative survival rate was 97, 88, 80, 72, and 48%, respectively, in the low AGR group and 99, 96, 93, 85, and 79%, respectively, in the high AGR group. The Kaplan-Meier curves presented significant differences in all-cause mortality (log-rank = 24.84, *p* < 0.001) and cardiovascular mortality (log-rank = 14.02, *p* < 0.001) of the two AGR groups (Fig. 2). Furthermore, when considering drop out due to other causes as competitive risks, the risk of cumulative mortality incidence was significantly higher in patients in the low AGR group than in the high AGR group (Gray test 25.28, *p* < 0.001) (Fig. 3).

The association between AGR level and all-cause mortality and cardiovascular mortality are listed in Table 3. In crude analysis, the patients with low AGR had a significantly increased risk of all-cause and cardiovascular mortality [Hazard Ratio (HR): 2.50, 95% Confidence interval (CI): 1.72–3.63, *p* < 0.001 and HR: 2.88, 95% CI: 1.62–5.14, *p* < 0.001 respectively]. After adjustment for potential confounders, low AGR level was still associated with an increased all-cause and CVD mortality [HR: 1.57, 95% CI: 1.07–2.32, *p* = 0.022 and HR: 2.01, 95% CI: 1.10–3.69, *p* = 0.023, respectively].

**Table 3:** Association between baseline AGR and all-cause and cardiovascular mortality

	All-cause mortality		Cardiovascular mortality	
	HR(95%CI)	*p*	HR(95%CI)	*p*
Unadiusted				
AGR ≤ 1.25 vs	2.50 (1.72–3.63)	< 0.001	2.88 (1.62–5.14)	< 0.001
AGR > 1.26	1		1	
Model 1^a^				
AGR ≤ 1.25 vs	1.80 (1.24–2.62)	0.002	2.18 (1.22–3.91)	0.009
AGR > 1.26	1		1	
Model 2^b^				
AGR ≤ 1.25 vs	1.57 (1.07–2.32)	0.022	2.01 (1.10–3.69)	0.023
AGR > 1.26	1		1	

Abbreviations: *AGR* = Albumin-globulin ratio, *HR* = Hazard ratio, *CCI* = Charlson comorbidity index, *RDW* = Red cell distribution width, 95%*CI* = 95% Confidence interval

^a^ Adjusted for sex and CCI

^b^Adjusted for model1 covariates and leukocytes, hemoglobin, platelet, RDW, phosphorus, albumin-corrected calcium, serum creatinine, urea nitrogen, serum uric acid, peritoneal dialysate glucose concentration

## Discussion

In this respectively study of 602 adult incident PD patients, we found that lower AGR levels were associated with comorbidities, impaired immunonutritional status, and prothrombotic status, including lower serum albumin, higher leukocytes, higher creatinine, higher urea nitrogen, higher platelet count, and higher RDW. Compared to patients with AGR > 1.26, the all-cause and CVD mortality risks were increased in patients with an AGR ≤ 1.25 even after adjusting for potential confounders. Therefore, AGR may be a useful parameter in identifying patients at risk of mortality.

The association of AGR and mortality had been reported by previous studies on varied populations, including non-ST elevation myocardial infarction [19], colorectal cancer [20, 21], hepatocellular carcinoma [22], and early-stage non-small cell lung cancer [23]. Suh et al. reported low AGR was a risk factor for cancer incidence and mortality, both in the short- and long terms, in 26,974 healthy patients [24]. In this study, we demonstrated that lower AGR was associated with higher all-cause and CVD mortality in PD patients. These data indicated that AGR could be a risk factor for all-cause and CVD mortality in PD patients.

Significant older age, higher prevalence of diabetes, and higher level of CCI have been found in patients with lower AGR, which suggests an association of AGR with traditional mortality risk, including disease burden. These findings are consistent with the previous studies [19, 25]. Beamer et al. found diabetes was more common in patients with lower AGR and presumed low AGR as a marker of severity of the pathophysiological changes related to chronic disease, such as diabetes [25]. In additional, Beamer et al. noted that acute stroke patients with a low AGR appeared to experience a preponderance of recurrent vascular events [25]. A low AGR could be the consequence of transcapillary albumin loss [26], such as proteinuria. Proteinuria was associated with an increased risk for cardiovascular events and acute cerebrovascular disease, even in the general population [27, 28]. Therefore, patients with low AGR may indicate vascular damage.

This study revealed that AGR negatively correlated with leukocytes, which indicated that the higher risk of mortality in the low AGR group might be partly due to inflammation. Proinflammatory cytokines were inversely correlated with serum albumin in patients treated with peritoneal dialysis, suggesting hypoalbuminemia could be regarded as a marker of inflammation in dialysis patients [29, 30]. Serum albumin in PD patients is always lower than healthy people, and the results present 428 patients had a level of albumin < 35 g/L. It was reported that a low albumin level was associated with increased PD mortality [31, 32]. Furthermore, Filipa et al. found that ESRD patients who had a low albumin level and high C-reactive protein (CRP) had a significantly higher mortality. However, the mortality of those with a low albumin and normal CRP level did not increase, which indicated the increased mortality of hypoalbuminemia was partly attributed to inflammation [33]. In addition, this study showed that baseline globulin levels were higher in the low AGR group, and it could be interpreted by the increase of some acute phase proteins which indicated an inflammatory status in the PD patients. Serum IL-6 levels were higher in PD patients than healthy persons [34], and plays a major role in the production of most acute phase proteins, such as CRP, fibrinogen, and so on [35], which were important constituents of non-albumin proteins.

Numerous studies confirmed malnutrition was associated with all-cause mortality in PD patients [36, 37]. This study showed that AGR was positively correlated with some interrelated prognostic factors for malnutrition, including serum albumin, creatinine, and phosphorus, and negatively correlated with RDW, which has been demonstrated to be data reflecting malnutrition and inflammation, associated with an increased CVD mortality in PD patients in our previous study [38]. As mentioned above, AGR may serve as an index of immunonutritional in PD patients.

Shifting to a prothrombotic environment might be another possibile mechanism responsible for the association between AGR and mortality. Tanahashi et al. found that a lower AGR was correlated with an increase in erythrocyte aggregability in cerebrovascular disease [39]. Acute stroke patients with a low AGR appeared to experience a preponderance of recurrent vascular events. This suggested lower AGR represents a prothrombotic environment, in line with the findings reported herein. This study found a lower AGR was associated with a higher platelet count, which plays a key role in thrombosis, including erythrocyte aggregability, and was a risk factor of cardiovascular mortality in incident PD patients in our previous study [40]. A lower AGR was associated with higher RDW, which suggests impaired blood flow, indicating prothrombotic.

There are some limitations to this study. Firstly, the confidence intervals in multivariate Cox regression analysis were wide, this might due to a relatively small sample because of a retrospective, single-center study. However, we performed a Cox regression power analysis, and the power of AGR based on our pragmatically obtained sample was > 99% and > 99% for the Cox regression model of all-cause and cardiovascular mortality, respectively. Secondly, due to the limitations of certain conditions, we had no available data of some specific inflammatory markers, such as cytokines. Finally, we only investigated the association of baseline AGR level and PD mortality. Changes of AGR over time, requires further study. We have excluded some potential bias caused by treatment modality. Patients on PD for the 2nd or 3rd time were excluded from this study. Exposure to dialysate over time would lead to hyperpermeability of peritoneal capillaries, resulting in a potential bias [41]. PD patients who were transferred from HD were also excluded. The main reason for the transfer from HD to PD is because of cardiovascular disease [42], which is a risk factor of PD mortality. After a period on HD, most patients lose their residual renal function (RRF) and in these adequate solute clearances and ultrafiltration could be difficult to achieve with PD. Additionally, patients who transferred from HD to PD had a higher rate of peritonitis, PD technique failure, and death [43, 44]. The association between AGR and patient’s survival on HD needs to be explored in the future. More multi-center prospective studies including more patients are required to evaluate these findings.

## Conclusion

In conclusion, a lower AGR was associated with higher CVD and all-cause mortality in PD patients, independently. Our results suggest that AGR may be a useful index in identifying patients on PD who are at risk for CVD and all-cause mortality.

## Data Availability

The datasets used and/or analyzed during the current study are available from the corresponding author on reasonable request.

## Abbreviations

AGR: Albumin-globulin ratio
CCI: Charlson comorbidity index
CI: Confidence interval
CRP: C-reactive protein
CVD: Cardiovascular disease
ESRD: End-stage renal disease
HD: Hemodialysis
HR: Hazard ratio
PD: Peritoneal dialysis
PEW: Protein-energy wasting
RDW: Red cell distribution width
RRF: Residual renal function

## References

[1] Saran R, Robinson B, Abbott KC, et al. US Renal Data System 2018 Annual Data Report: Epidemiology of Kidney Disease in the United States. Am J Kidney Dis, ll. 2019;73(3S1):A7. 10.1053/j.ajkd.2019.01.001 PMC6620109 30798791

[2] Ho YW, Chau K, Choy BY (2013). Hong Kong renal registry report 2012. Hong Kong J Nephrol.

[3] Zhang H, Zhang B, Zhu K (2019). Preoperative albumin-to-globulin ratio predicts survival in patients with non-small-cell lung cancer after surgery. J Cell Physiol.

[4] Huang CY, Liou SY, Kuo WW, Wu HC, Chang YL, Chen TS (2016). Chemiluminescence analysis of antioxidant capacity for serum albumin isolated from healthy or uremic volunteers. Luminescence.

[5] Kaysen GA, Dubin JA, Muller HG, Mitch WE, Rosales LM, Levin NW (2002). Relationships among inflammation nutrition and physiologic mechanisms establishing albumin levels in hemodialysis patients. Kidney Int.

[6] Han SH, Han DS (2012). Nutrition in patients on peritoneal dialysis. Nat Rev Nephrol.

[7] Peng F, Chen W, Zhou W (2017). Low prognostic nutritional index associated with cardiovascular disease mortality in incident peritoneal dialysis patients. Int Urol Nephrol.

[8] Parija SC (2012). Textbook of Microbiology & Immunology.

[9] Liu YL, Liu JH, Wang IK (2017). Association of inflammatory cytokines with mortality in peritoneal dialysis patients. Biomed (Taipei).

[10] Liu SH, Chen CY, Li YJ (2017). The value of time-averaged serum high-sensitivity C-reactive protein in prediction of mortality and dropout in peritoneal dialysis patients. Ther Clin Risk Manag.

[11] Bozkaya Y, Erdem GU, Demirci NS (2019). Prognostic importance of the albumin to globulin ratio in metastatic gastric cancer patients. Curr Med Res Opin.

[12] Zhou T, Yu ST, Chen WZ, Xie R, Yu JC (2019). Pretreatment albumin globulin ratio has a superior prognostic value in laryngeal squamous cell carcinoma patients: a comparison study. J Cancer.

[13] Ahn SS, Yoo J, Jung SM, Song JJ, Park YB, Lee SW (2019). Clinical role of albumin to globulin ratio in microscopic polyangiitis: a retrospective monocentric study. Clin Rheumatol.

[14] Li K, Fu W, Bo Y, Zhu Y (2018). Effect of albumin-globulin score and albumin to globulin ratio on survival in patients with heart failure: a retrospective cohort study in China. BMJ Open.

[15] American DA (2019). 2. Classification and diagnosis of diabetes: standards of medical Care in Diabetes-2019. Diab Care.

[16] Fried L, Bernardini J, Piraino B (2001). Charlson comorbidity index as a predictor of outcomes in incident peritoneal dialysis patients. Am J Kidney Dis.

[17] Wen Y, Guo Q, Yang X (2015). High glucose concentrations in peritoneal dialysate are associated with all-cause and cardiovascular disease mortality in continuous ambulatory peritoneal dialysis patients. Perit Dial Int.

[18] Benjamin EJ, Virani SS, Callaway CW (2018). Heart disease and stroke Statistics-2018 update: a report from the American Heart Association. Circ.

[19] Azab B, Bibawy J, Harris K (2013). Value of albumin-globulin ratio as a predictor of all-cause mortality after non-ST elevation myocardial infarction. Angiol.

[20] Azab B, Kedia S, Shah N (2013). The value of the pretreatment albumin/globulin ratio in predicting the long-term survival in colorectal cancer. Int J Color Dis.

[21] Shibutani M, Maeda K, Nagahara H (2015). The pretreatment albumin to globulin ratio predicts chemotherapeutic outcomes in patients with unresectable metastatic colorectal cancer. BMC Cancer.

[22] Deng Y, Pang Q, Miao RC (2016). Prognostic significance of pretreatment albumin/globulin ratio in patients with hepatocellular carcinoma. Onco Targets Ther.

[23] Wang Y, Li S, Hu X (2019). The prognostic value of serum albumin-globulin ratio in early-stage non-small cell lung cancer: a retrospective study. Cancer Manag Res.

[24] Suh B, Park S, Shin DW (2014). Low albumin-to-globulin ratio associated with cancer incidence and mortality in generally healthy adults. Ann Oncol.

[25] Beamer N, Coull BM, Sexton G, de Garmo P, Knox R, Seaman G (1993). Fibrinogen and the albumin-globulin ratio in recurrent stroke. Stroke.

[26] Fleck A, Raines G, Hawker F (1985). Increased vascular permeability: a major cause of hypoalbuminaemia in disease and injury. Lancet.

[27] Wang A, Chen G, Cao Y (2017). Estimated glomerular filtration rate, proteinuria, and risk of cardiovascular diseases and all-cause mortality in diabetic population: a community-based cohort study. Sci Rep.

[28] Wang A, Liu X, Su Z, et al. Two-Year Changes in Proteinuria and the Risk of Stroke in the Chinese Population: A Prospective Cohort Study. J Am Heart Assoc. 2017;6(7):e006271 10.1161/JAHA.117.006271 PMC5586318 28666989

[29] Milan Manani S, Virzi GM, Clementi A (2016). Pro-inflammatory cytokines: a possible relationship with dialytic adequacy and serum albumin in peritoneal dialysis patients. Clin Kidney J.

[30] Shioya M, Yoshida T, Kasai K (2013). Inflammatory factors for hypoalbuminemia in Japanese peritoneal dialysis patients. Nephrol (Carlton).

[31] Chiu PF, Tsai CC, Wu CL (2016). Trajectories of serum albumin predict survival of peritoneal Dialysis patients: a 15-year follow-up study. Medicine (Baltimore).

[32] Mehrotra R, Duong U, Jiwakanon S (2011). Serum albumin as a predictor of mortality in peritoneal dialysis: comparisons with hemodialysis. Am J Kidney Dis.

[33] Alves FC, Sun J, Qureshi AR (2018). The higher mortality associated with low serum albumin is dependent on systemic inflammation in end-stage kidney disease. PLoS One.

[34] Borazan A, Ustun H, Ustundag Y (2004). The effects of peritoneal dialysis and hemodialysis on serum tumor necrosis factor-alpha, interleukin-6, interleukin-10 and C-reactive-protein levels. Mediat Inflamm.

[35] Castell JV, Gomez-Lechon MJ, David M, Fabra R, Trullenque R, Heinrich PC (1990). Acute-phase response of human hepatocytes: regulation of acute-phase protein synthesis by interleukin-6. Hepatol.

[36] Leinig CE, Moraes T, Ribeiro S (2011). Predictive value of malnutrition markers for mortality in peritoneal dialysis patients. J Ren Nutr.

[37] Yan X, Yang X, Xie X (2017). Association between comprehensive nutritional scoring system (CNSS) and outcomes of continuous ambulatory peritoneal Dialysis patients. Kidney Blood Press Res.

[38] Peng F, Li Z, Zhong Z (2014). An increasing of red blood cell distribution width was associated with cardiovascular mortality in patients on peritoneal dialysis. Int J Cardiol.

[39] Tanahashi N, Gotoh F, Tomita M (1989). Enhanced erythrocyte aggregability in occlusive cerebrovascular disease. Stroke.

[40] Pen, Li Z, Yi C (2017). Platelet index levels and cardiovascular mortality in incident peritoneal dialysis patients: a cohort study. Platelets..

[41] Shi Y, Xiong Y, Lei Y (2019). Protective effect of COMP-angiopoietin-1 on peritoneal vascular permeability and peritoneal transport function in uremic peritoneal dialysis rats. Am J Transl Res.

[42] Van Biesen W, Dequidt C, Vijt D, Vanholder R, Lameire N (1998). Analysis of the reasons for transfers between hemodialysis and peritoneal dialysis and their effect on survivals. Adv Perit Dial.

[43] Nguyen ANL, Prasad Kafle M, Sud K, Lee VW (2019). Predictors and outcomes of patients switching from maintenance haemodialysis to peritoneal dialysis in Australia and New Zealand: strengthening the argument for 'peritoneal dialysis first' policy. Nephrol (Carlton).

[44] Nessim SJ, Bargman JM, Jassal SV, Oliver MJ, Na Y, Perl J (2015). The impact of transfer from hemodialysis on peritoneal dialysis technique survival. Perit Dial Int.

